# Comparison between traditional logistic regression and machine learning for predicting mortality in adult sepsis patients

**DOI:** 10.3389/fmed.2024.1496869

**Published:** 2025-01-06

**Authors:** Hongsheng Wu, Biling Liao, Tengfei Ji, Keqiang Ma, Yumei Luo, Shengmin Zhang

**Affiliations:** Hepatobiliary Pancreatic Surgery Department, Huadu District People’s Hospital of Guangzhou, Guangzhou, China

**Keywords:** machine learning, random forest, logistic regression, adult sepsis, mortality

## Abstract

**Background:**

Sepsis is a life-threatening disease associated with a high mortality rate, emphasizing the need for the exploration of novel models to predict the prognosis of this patient population. This study compared the performance of traditional logistic regression and machine learning models in predicting adult sepsis mortality.

**Objective:**

To develop an optimum model for predicting the mortality of adult sepsis patients based on comparing traditional logistic regression and machine learning methodology.

**Methods:**

Retrospective analysis was conducted on 606 adult sepsis inpatients at our medical center between January 2020 and December 2022, who were randomly divided into training and validation sets in a 7:3 ratio. Traditional logistic regression and machine learning methods were employed to assess the predictive ability of mortality in adult sepsis. Univariate analysis identified independent risk factors for the logistic regression model, while Least Absolute Shrinkage and Selection Operator (LASSO) regression facilitated variable shrinkage and selection for the machine learning model. Among various machine learning models, which included Bagged Tree, *Boost Tree, Decision Tree*, *LightGBM*, *Naïve Bayes*, *Nearest Neighbors*, *Support Vector Machine (SVM)*, and *Random Forest (RF)*, the one with the maximum area under the curve (AUC) was chosen for model construction. Model validation and comparison with the Sequential Organ Failure Assessment (SOFA) and the Acute Physiology and Chronic Health Evaluation (APACHE) scores were performed using receiver operating characteristic (ROC) curves, calibration curves, and decision curve analysis (DCA) curves in the validation set.

**Results:**

Univariate analysis was employed to assess 17 variables, namely gender, history of coronary heart disease (CHD), systolic pressure, white blood cell (WBC), neutrophil count (NEUT), lymphocyte count (LYMP), lactic acid, neutrophil-to-lymphocyte ratio (NLR), red blood cell distribution width (RDW), interleukin-6 (IL-6), prothrombin time (PT), international normalized ratio (INR), fibrinogen (FBI), D-dimer, aspartate aminotransferase (AST), total bilirubin (Tbil), and lung infection. Significant differences (*p* < 0.05) between the survival and non-survival groups were observed for these variables. Utilizing stepwise regression with the “backward” method, independent risk factors, including systolic pressure, lactic acid, NLR, RDW, IL-6, PT, and Tbil, were identified. These factors were then incorporated into a logistic regression model, chosen based on the minimum Akaike Information Criterion (AIC) value (98.65). Machine learning techniques were also applied, and the RF model, demonstrating the maximum Area Under the Curve (AUC) of 0.999, was selected. LASSO regression, employing the lambda.1SE criteria, identified systolic pressure, lactic acid, NEUT, RDW, IL6, INR, and Tbil as variables for constructing the RF model, validated through ten-fold cross-validation. For model validation and comparison with traditional logistic models, SOFA, and APACHE scoring.

**Conclusion:**

Based on deep machine learning principles, the RF model demonstrates advantages over traditional logistic regression models in predicting adult sepsis prognosis. The RF model holds significant potential for clinical surveillance and interventions to enhance outcomes for sepsis patients.

## Introduction

Sepsis represents a critical condition marked by organ dysfunction resulting from an imbalanced host response to infection, leading to high mortality rates and substantial healthcare costs (1, 2). Despite the establishment of the initial consensus definitions (Sepsis-1) in 1991, the global incidence of sepsis continues to rise, making the true epidemiology of sepsis a subject of ongoing concern. Further exploration of high-risk factors associated with sepsis-related mortality is essential (3). In clinical settings, the evaluation of sepsis severity and the identification of risk factors for mortality often rely on scoring systems such as Sequential Organ Failure Assessment (SOFA), Quick Sequential Organ Failure Assessment (qSOFA), and Acute Physiology and Chronic Health Evaluation (APACHE) (4–6). However, these scoring systems involve numerous parameters, posing challenges for clinical practitioners. Consequently, there has been a growing interest in exploring the effectiveness of biomarkers and clinical prediction models in predicting the prognosis of sepsis patients (7–9).

Over the past few years, linear regression models have dominated the clinical landscape for predicting sepsis mortality (10, 11). However, their limitations, including the inability to handle non-linearity among variables, sensitivity to outlier values, and the need to meet the linear regression hypothesis, constrain their utility with non-linear and imbalanced datasets (12). Machine learning (ML) is a subfield of artificial intelligence (AI) that focuses on developing systems capable of learning from data or improving performance. Specifically, machine learning is a technique that enables computers to create models by training algorithms using datasets (13). Previous studies had indicated that ML models play a crucial role in predicting the prognosis of sepsis patients. ML models had demonstrated superior predictive accuracy compared to traditional statistical methods. By leveraging complex algorithms, these models can identify non-linear relationships and interactions between variables that may be overlooked by simpler models, leading to more precise predictions of sepsis mortality (14, 15). On the other hand, the key strengths of ML models is their ability to handle high-dimensional data effectively. They can incorporate a vast array of clinical variables, which allows for a more comprehensive understanding of the patient’s condition and the factors contributing to mortality risk (16, 17). Consequently, these above benefits position ML as an essential tool in the prediction of sepsis mortality, aiding in the improvement of clinical decision-making and patient outcomes.

Based on the methodological review mentioned above, we employed both traditional generalized linear regression and ML models to assess their predictive capabilities in adult sepsis’s mortality during their hospitalization duration. Notably, we conducted internal validation for both models and compared their performance with SOFA and APACHE scores in terms of discrimination, calibration, and clinical practicality. This comprehensive analysis offers profound insights into mortality risk adjustment for observational adult sepsis datasets, contributing valuable information to the understanding of predictive models and their applicability in clinical settings. This study closely complied with TRIPOD guidelines (18) and the PROBAST risk of bias tool (19).

## Methods

### Source of clinical data

The clinical data for this cross-sectional study were obtained from the electronic medical records of Huadu District People’s Hospital of Guangzhou, Southern Medical University. The study focused on adult patients diagnosed with sepsis during hospitalization from January 2020 to December 2022, adhering to the Sepsis-3 definition (20, 21). Exclusion criteria included patients under 18 years old, those with malignant tumors, individuals with immunosuppression, those who died or withdrew treatment within 24 h of admission, and cases where clinical data could not be extracted. Following these criteria, 606 cases of adult sepsis were included in the study. Due to it directly reflects the sepsis patient’s survival and is a key performance indicator during the hospitalization duration, we define the mortality as the outcome of this study.

### Variables extraction

Variable extraction involved retrieving general information (gender, age, and body mass index), medical history [hypertension, diabetes, and coronary heart disease (CHD)], clinical signs (temperature, heart rate, systolic pressure, and infection site), laboratory examination results [white blood cell count (WBC), platelet count, neutrophil (NEUT) and lymphocyte (LYMP) counts, neutrophil-to-lymphocyte ratio (NLR), red cell distribution width (RDW), C-reactive protein, procalcitonin, lactic acid, prothrombin time (PT), international normalized ratio (INR), fibrinogen (FIB), D-dimer, creatinine, alanine transaminase (ALT), aspartate transaminase (AST), total bilirubin (Tbil), and interleukin-6 (IL-6)], etiologic detection (Gram-positive bacteria, Gram-negative bacteria, or fungal), and severity scores of sepsis (SOFA and APACHE) from the electronic medical record system. All data were extracted within the first 24 h of patient admission. For missing values, multiple imputation was performed using the “*mice*” package in R software.

### Model construction of logistic regression

The study divided the 606 adult sepsis cases randomly into a training set (*n* = 435) and a validation set (*n* = 171) at a ratio of 7:3. Based on whether the patient died or not between 24 h after admission and discharge, participants were categorized into a survival group (421) and a non-survival group (185). For traditional logistic regression model construction, univariate analysis identified significant risk factors (*p* < 0.05), which were then included in binary logistic regression. The stepwise regression with the “backward” method was employed to achieve the optimal model with the least AIC value.

### Machine learning model selection and construction

For ML model selection, eight integrated algorithms, including *Bagged Tree*, *Boost Tree*, *Decision Tree*, *LightGBM*, *Naïve Bayes*, *Nearest Neighbors*, *Support SVM*, and *RF*, were considered. In the “*tidymodels*” framework of R software, workflow sets were used to compare these models, perform resampling, and tune parameters. Because of its ability to provide a comprehensive measure of a model’s performance across all classification thresholds, we select AUC as an optimum index in order to offer more nuanced view of model performance. The ML model with the highest AUC value was chosen for model construction. In order to perform variable shrinkage and selection, which may avoid the overfitting of the ML model, we utilized LASSO regression with ten-fold cross-validation. The count of variables in the ultimate model was ascertained based on the specific location of lambda.1SE, a coefficient that signifies the ideal equilibrium between model intricacy and forecasting accuracy.

### Models validation and comparison

**Figure 1:**
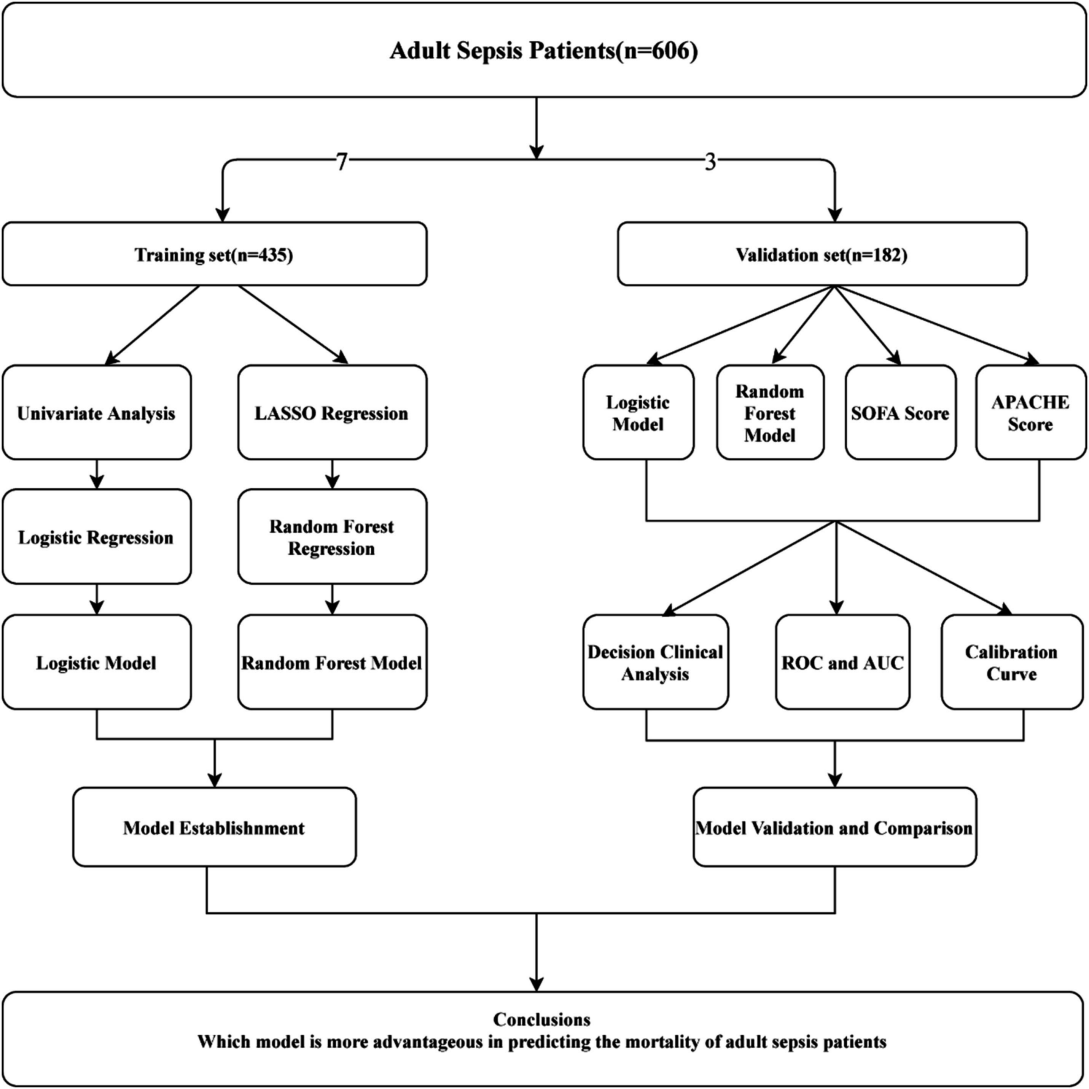
Flowchart illustrating the research design.

Models were validated and compared using discrimination, calibration, clinical benefit, and generalization. Discrimination was assessed by calculating the AUC of the ROC, while calibration was evaluated using calibration curves and the Hosmer-Lemeshow test. Decision curve analysis (DCA) curves were employed to assess the clinical benefit of the models. To estimate generalization, logistic regression, and ML models were compared with SOFA and APACHE scores using discrimination, calibration, and DCA for both the training and validation sets. The research design flowchart is depicted in Figure 1.

### Variables importance

During traditional logistic regression, the importance of variables is determined by assessing the absolute value of each regression coefficient from the covariate. A larger absolute value indicates a more significant and important predictor. Variable importance is a key characteristic of ML models. In ML models, if changing the value of a variable leads to false prediction results, it implies that the variable is sensitive to classification outcomes and holds greater importance. The calculation of variable importance in the ML model such as RF involves determining the importance of each single decision tree, and by considering the number of trees set in the RF, the average of these values yields the overall variable importance of the RF model (22, 23).

### Ethics statement

Data extraction and collection for this study were approved by the Ethics Committee of Huadu District People’s Hospital of Guangzhou (Registration Number: 2023088). Due to the retrospective nature of the study, the Ethics Committee of Huadu District People’s Hospital of Guangzhou waived the need of obtaining informed consent. And we had confirmed that the method of this research was performed in accordance with the regulation of Ethics Committee of Huadu District People’s Hospital of Guangzhou.

### Statistical analysis

R version 4.1.3 was used for data analysis and the creation of statistical figures. Missing values in this cross-sectional study were addressed through multiple imputations using the “*mice*” package. The study population of adult sepsis patients was divided into training and validation sets using the “*caret*” package. Descriptive statistics, including mean ± standard deviation for continuous data with normal distribution and median (upper and lower quartiles) for non-normally distributed data, were employed to characterize average values. For univariate analysis, the Chi-square test was used to analyze differences in categorical data, while *t*-tests and Mann–Whitney *U* tests were employed for normally and non-normally distributed continuous data, respectively.

For logistic regression model construction, the “*glm*” function was used to conduct univariate analysis and binary logistic regression. The final model was determined by stepwise regression with the least AIC value using the “backward” method. For machine learning models, the Least Absolute Shrinkage and Selection Operator (LASSO) regression was utilized for variable shrinkage and selection, and the “*glmnet*” package was employed with parameter tuning using lambda.1SE under ten-fold cross-validation to remove irrelevant variables. The framework of “*tidymodels*” facilitated model selection, construction, workflow settings, validation, and comparison of predictive capabilities. Random forest was selected as the machine learning model based on the highest AUC and accuracy values, and the “*randomForest*” package was used to fit the model. To optimize the out-of-bag (OOB) error and improve predictive efficacy, the “*tuneRF*” function was employed.

Discrimination of the models was investigated using ROC and AUC with the “*pROC*” package. Calibration was assessed using the “*calibration*” function from the “*rms*” package, and the Hosmer-Lemeshow test was performed. Net benefits, reflecting model clinical practicality, were calculated and compared using DCA with the “*ggDCA*” package.

## Results

### Baseline analysis and data splitting

The study included a total of 606 patients diagnosed with sepsis, categorized into a survival group (*n* = 421) and a non-survival group (*n* = 185) based on hospital stay duration. To facilitate model construction and validation, a random allocation resulted in a training set (*n* = 435) and a validation set (*n* = 171) at a 7:3 ratio. Details of the baseline analysis and data splitting are presented in Table 1.

**Table 1:** Baseline analysis and data splitting.

Characteristics		All patients (*n* = 606)	Survival (*n* = 421)	Non-survival (*n* = 185)	Training set (*n* = 435)	Validation set (*n* = 171)	*p*-value
Gender							0.914
Female	*n* (%)	269 (44.4%)	207(49.2%)	62(33.5%)	192 (44.1%)	77 (45.0%)	
Male	*n* (%)	337 (55.6%)	214(50.8%)	123(66.5%)	243 (55.9%)	94 (55.0%)	
Age	* $\bar{x}$ ± sd	64.0 ± 17.5	63.6 ± 16.5	64.9 ± 17.6	64.7 ± 18.2	63.2 ± 15.8	0.163
Infection site							0.806
Respiratory system	*n* (%)	232 (38.3%)	132(31.4%)	100(54.1%)	166 (38.2%)	66 (38.6%)	
Urinary system	*n* (%)	161 (26.6%)	100(23.6%)	61(33.0%)	113 (26.0%)	48 (28.1%)	
Digestive system	*n* (%)	213 (35.1%)	189(45.0%)	24(12.9%)	156 (35.9%)	57 (33.3%)	
Pathology							0.332
Gram-positive	*n* (%)	331 (54.6%)	223(53.0%)	108(58.4%)	230 (52.9%)	101 (59.1%)	
Gram-negative	*n* (%)	252 (41.6%)	180(42.8%)	72(38.9%)	189 (43.4%)	63 (36.8%)	
Fungal	*n* (%)	23 (3.80%)	18(4.2%)	5(2.7%)	16 (3.68%)	7 (4.09%)	
Diabetes							0.632
No	*n* (%)	411 (67.8%)	288(68.4%)	123(66.5%)	298 (68.5%)	113 (66.1%)	
Yes	*n* (%)	195 (32.2%)	133(31.6%)	62(33.5%)	137 (31.5%)	58 (33.9%)	
Hypertension							0.193
No	*n* (%)	360 (59.4%)	262(62.2%)	98(53.0%)	266 (61.1%)	94 (55.0%)	
Yes	*n* (%)	246 (40.6%)	159(37.8%)	87(47.0%)	169 (38.9%)	77 (45.0%)	
CHD							0.304
No	*n* (%)	390 (64.4%)	298(70.8%)	92(49.7%)	274 (63.0%)	116 (67.8%)	
Yes	*n* (%)	216 (35.6%)	123(29.2%)	93(50.3%)	161 (37.0%)	55 (32.2%)	
BMI	$\bar{x}$ ± sd	24.3 ± 4.6	24.3 ± 4.6	24.3 ± 4.7	24.2 ± 4.7	23.9 ± 4.5	0.693
Systolic pressure (mmHg)	$\bar{x}$ ± sd	124 ± 23.5	131.1 ± 18.0	93.66 ± 24.7	124.8 ± 19.2	124.6 ± 20.3	0.907
Heart rate (time/min)	$\bar{x}$ ± sd	122 ± 9.6	116.9 ± 11.58	118.1 ± 9.1	122 ± 8.8	121 ± 10.2	0.887
Temperature (°C)	$\bar{x}$ ± sd	38.5 ± 0.7	38.3 ± 0.7	38.3 ± 0.6	38.3 ± 0.6	38.5 ± 0.5	0.387
WBC (×10^9^/L)	*M[P_25_;P_75_]	12.7 [7.6;18.2]	11.9 [7.5;16.1]	15.0 [8.2;23.4]	12.9 [8.1;18.3]	11.8 [6.9;17.6]	0.107
Platelet (×10^9^/L)	M[P_25_;P_75_]	172 [111;249]	180.0 [125.0;252.0]	150.0 [63.0;242.5]	172 [114;246]	169 [95.5;263]	0.715
NEUT (×10^9^/L)	M[P_25_;P_75_]	10.6 [6.2;16.0]	9.8 [5.9;14.2]	14.1 [7.6;20.6]	10.7 [6.54;16.2]	9.85 [5.35;15.2]	0.149
LYMP (×10^9^/L)	M[P_25_;P_75_]	0.85 [0.5;1.4]	1.0 [0.7;1.5]	0.5 [0.3;0.6]	0.86 [0.5;1.4]	0.85 [0.5;1.4]	0.973
NLR	M[P_25_;P_75_]	12.5 [6.6;22.1]	10.3 [5.5;16.0]	26.2 [13.0;46.5]	12.4 [6.8;22.8]	12.7 [5.9;20.4]	0.431
RDW	$\bar{x}$ ± sd	44.7 ± 8.4	45.2 ± 8.2	50.5 ± 10.5	44.3 ± 8.7	45.2 ± 9.4	0.368
CRP (mg/ml)	M[P_25_;P_75_]	117 [47.6;179]	112.3 [40.5;166.7]	135.0 [56.9;194.2]	119 [46.6;179]	117 [54.9;176]	0.957
PCT (ng/ml)	M[P_25_;P_75_]	12.0 [2.42;41.1]	9.1 [1.6;36.0]	18.0 [6.3;53.5]	12.0 [2.49;40.7]	12.0 [2.20;39.5]	0.879
Lactic acid (mmol/L)	M[P_25_;P_75_]	2.20 [1.5;4.4]	1.9 [1.4;2.6]	4.8 [3.1;8.7]	2.15 [1.6;4.2]	2.50 [1.5;5.3]	0.390
PT (S)	$\bar{x}$ ± sd	15.1 ± 2.4	14.9 ± 2.2	18.9 ± 8.8	15.1 ± 3.1	14.9 ± 3.4	0.811
INR	M[P_25_;P_75_]	1.19 [1.1;1.4]	1.2 [1.1;1.3]	1.3 [1.2;1.7]	1.19 [1.08;1.35]	1.17 [1.08;1.38]	0.634
FIB	$\bar{x}$ ± sd	4.8 ± 1.6	5.2 ± 1.8	4.4 ± 2.1	4.91 ± 1.7	4.6 ± 1.9	0.181
D dimer (ng/m)	M[P_25_;P_75_]	2,532 [1,230;4,955]	2,183 [1,190;4,197]	3,365 [1,597;7,461]	2,544 [1,233;5,239]	2,251 [1,232;4,607]	0.569
Creatinine (μmol/L)	M[P_25_;P_75_]	125 [74.0;246]	106.0 [70.0;214.8]	162.0 [92.5;289.0]	117 [72.6;234]	131 [79.3;272]	0.099
ALT (U/L)	M[P_25_;P_75_]	28.0 [16.5;52.0]	24.0 [15.7;43.1]	36.0 [20.1;74.0]	29.0 [16.7;52.6]	26.0 [15.7;49.9]	0.350
AST (U/L)	M[P_25_;P_75_]	31.0 [20.0;62.0]	26.0 [18.9;47.0]	57.1 [29.2;156.5]	31.0 [20.2;61.0]	31.5 [19.6;67.2]	0.859
Tbil (μmol/L)	M[P_25_;P_75_]	15.6 [10.2;24.6]	14.5 [9.9;21.9]	18.0 [10.9;34.2]	15.7 [10.3;25.0]	14.5 [9.28;23.4]	0.428
IL6 (pg/ml)	M[P_25_;P_75_]	3.70 [2.30;5.80]	3.0 [1.9;4.2]	6.5 [5.1;7.7]	3.70 [2.30;5.75]	3.70 [2.30;5.90]	0.696
SOFA	M[P_25_;P_75_]	6.1 [4.2;9.5]	5.0 [4.0;6.1]	12.0 [9.1;14.0]	6.00 [4.0;9.0]	6.0 [4.0;9.0]	0.369
APACHE	$\bar{x}$ ± sd	32.0 ± 8.1	29.2 ± 7.5	40.9 ± 9.1	31.0 ± 78.3	34.0 ± 8.6	0.052

* $\bar{x}$ ± sd: mean ± standard deviation; M[P_25_;P_75_]: M, median; P_25_, Lower quartile; P_75_, Upper quartile.

### Logistic regression model construction

We initially conducted univariate analysis for risk factor selection in the training set for the logistic regression model. The results of the univariate analysis revealed that with significant differences (*p* < 0.05) between the survival and non-survival groups for including variables, gender, CHD, systolic pressure, WBC, NEUT, LYMP, lactic acid, NLR, RDW, IL6, PT, INR, FBI, D-dimer, AST, Tbil, and lung infection were brought into multiple variable regression (Table 2). Based on these 17 variables, we utilized logistic step regression (backward step method) to optimize the model according to the Akaike Information Criterion (AIC). The results showed that with the least AIC value (98.65), the logistic model (OR: 1.012, 95% CI: 2.218–3.216) included systolic pressure, lactic acid, NLR, RDW, IL6, PT, and Tbil as the final determinants (Supplementary Table S1).

**Table 2:** Univariate analysis of risk factors between the survival group and non-survival group.

Characteristics	*B	*SE	*OR	95%CI	*Z*	*P*-value
Gender	0.699	0.303	2.01	1.11–3.65	2.308	0.021
Age	0.014	0.008	1.01	1–1.03	1.605	0.108
Diabetes	−0.142	0.336	0.87	0.45–1.68	−0.424	0.671
Hypertension	−0.016	0.308	0.98	0.54–1.8	−0.052	0.959
CHD	0.663	0.302	1.94	1.07–3.51	2.198	0.028
BMI	−0.033	0.033	0.97	0.91–1.03	−0.974	0.33
Systolic pressure	−0.104	0.014	0.9	0.88–0.93	−7.444	<0.001
Heart rate	0.001	0.016	1	0.97–1.03	0.091	0.928
Temperature	0.015	0.239	1.02	0.64–1.62	0.064	0.949
WBC	0.055	0.018	1.06	1.02–1.09	3.051	0.002
Platelet	−0.002	0.001	1	1–1.02	−1.643	0.1
NEUT	0.068	0.02	1.07	1.03–1.11	3.446	0.001
LYMP	−1.605	0.35	0.2	0.1–0.4	−4.586	<0.001
NLR	0.066	0.013	1.07	1.04–1.1	5.167	<0.001
RDW	0.071	0.018	1.07	1.04–1.11	4.016	<0.001
CRP	0.004	0.002	1	1–1.01	1.957	0.05
PCT	0.005	0.004	1.01	1–1.01	1.354	0.176
Lactic acid	0.496	0.084	1.64	1.39–1.94	5.929	<0.001
PT	0.529	0.093	1.7	1.41–2.04	5.686	<0.001
INR	2.751	0.613	15.65	4.71–52.05	4.49	<0.001
FIB	−0.172	0.079	0.84	0.72–0.98	−2.188	0.029
D dimer	0	0	1	1.23–2.45	4.438	<0.001
Creatinine	0.001	0.001	1	1–1.02	1.408	0.159
ALT	0.004	0.002	1	1–1.01	1.261	0.175
AST	0.005	0.001	1	1–1.01	3.198	0.001
Tbil	0.015	0.005	1.01	1–1.02	2.719	0.007
IL-6	0.728	0.102	2.07	1.7–2.53	7.157	<0.001
Infection site						
Respiratory system	1.839	0.325	6.29	3.33–11.89	5.664	<0.001
Urinary system	−0.17	0.349	0.84	0.43–1.67	−0.487	0.626
Digestive system	−1.941	0.395	0.14	0.75–1.95	−4.916	0.56
Pathology						
Gram-positive	0.235	0.303	1.26	0.7–2.29	0.774	0.439
Gram-negative	−0.14	0.309	0.87	0.47–1.59	−0.453	0.65
Fungal	−0.611	0.805	0.54	0.11–2.63	−0.759	0.448

*B: regression coefficient; SE: Standard Error; OR: Odds Ratio.

### Machine learning model and variables selection

**Figure 2:**
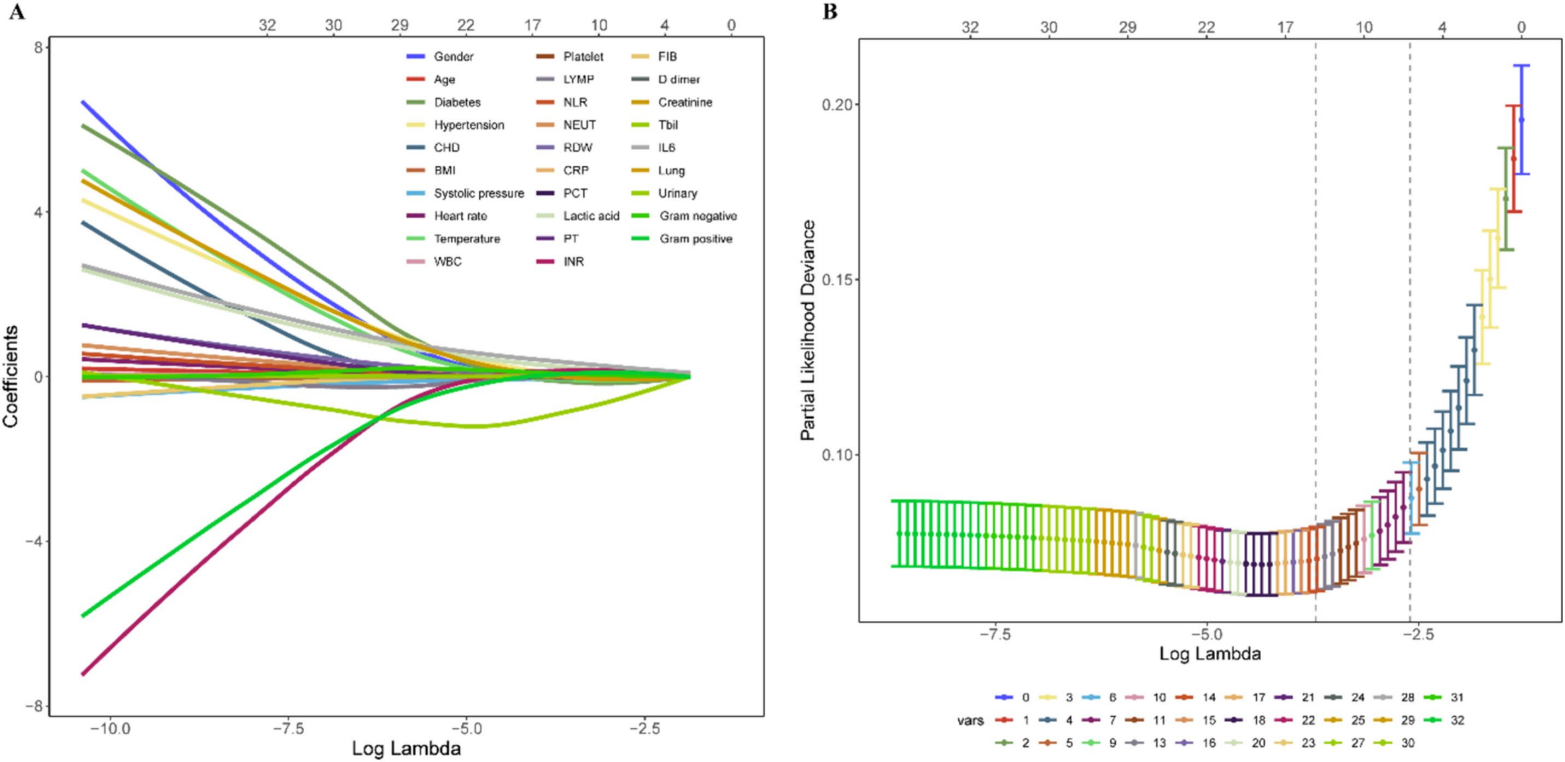
Variable shrinkage and selection by LASSO regression. **(A)** Shrinkage pathway of LASSO regression. **(B)** Based on ten-fold cross-validation, seven variables, including systolic pressure, lactic acid, NEUT, RDW, IL6, INR, and Tbil, were chosen using the lambda.1SE criteria.

**Figure 3:**
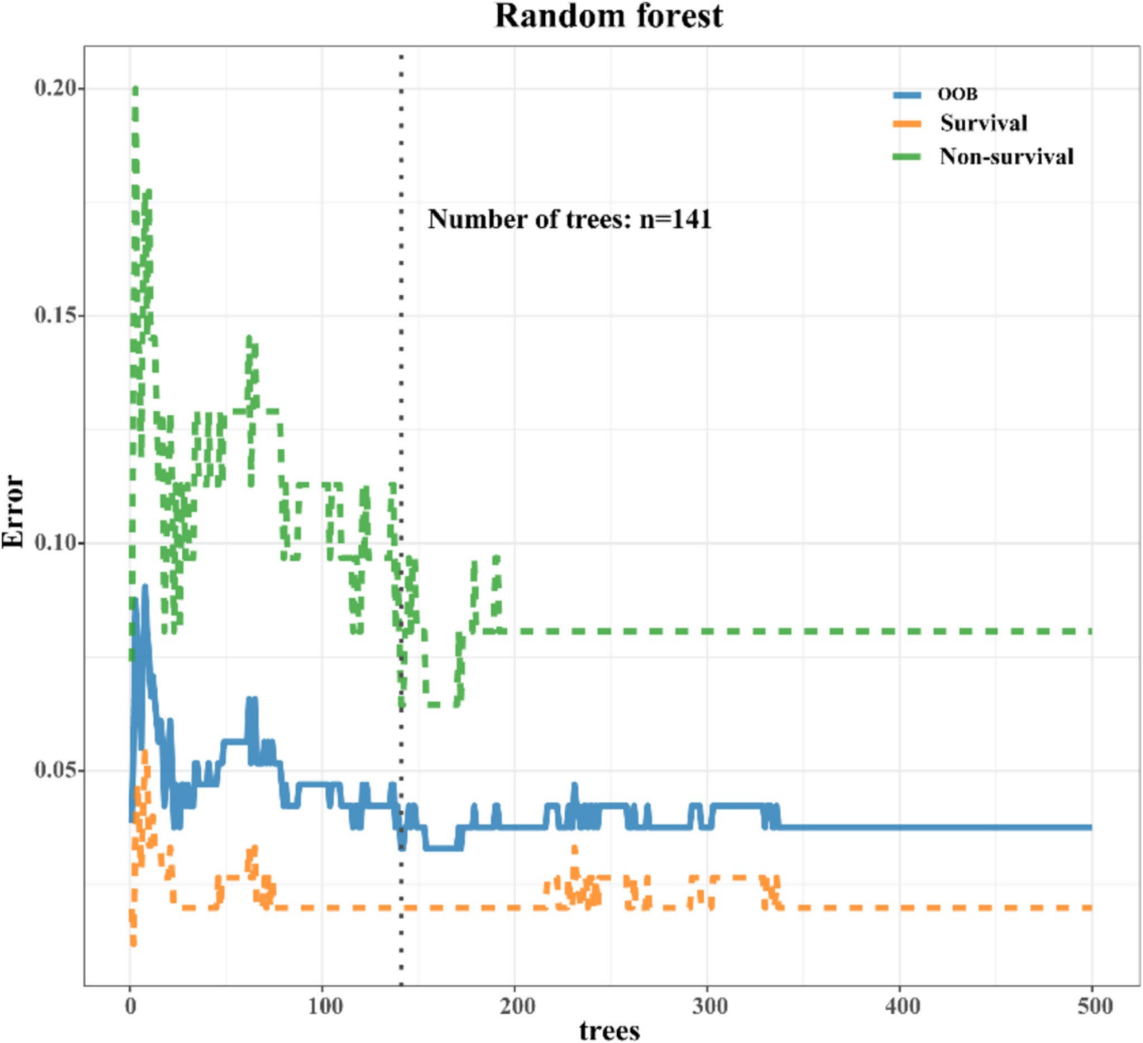
Error rate chart of RF model. As the iteration reached 141 decision trees, the error rates of both out-of-bag (OOB) and model classification showed a noticeable decrease, eventually reaching a steady state.

As depicted in Supplementary Figure S1, a comprehensive comparison of various machine learning models revealed that RF stood out with high accuracy and an AUC value of 0.99. Therefore, RF was chosen as the preferred methodology for model construction. Regarding variable selection, LASSO regression, employing ten-fold cross-validation with lambda.1SE criteria, identified systolic pressure, lactic acid, NEUT, RDW, IL6, INR, and Tbil as the chosen variables for RF model construction (Figure 2). During the RF modeling process, we initially set 500 decision trees for preliminary model calculation in the training set. To determine the optimal parameter for mortality prediction in sepsis patients, we utilized the OOB error as a measure of the model’s performance index. The results demonstrated that when the iteration reached 141 decision trees, the error rates of both OOB and model classification showed a noticeable decrease, reaching a stable state. This observation illustrated that the RF model achieved the most stable and optimal situation (Figure 3).

### Model validation and multi-models comparison

**Figure 4:**
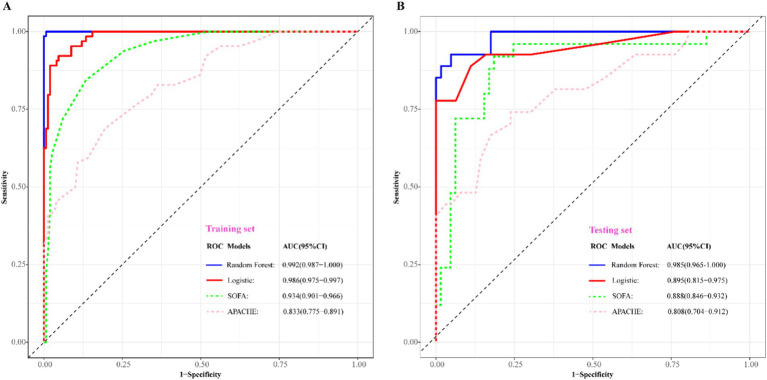
Comparison of discriminative ability among RF, logistic regression, SOFA, and APACHE scoring system. **(A)** Training set; **(B)** validation set. The blue solid ROC curves with the largest AUC values both in training set and validation set represented that RF associated with the best discrimination among the four models. AUC, area under curve; SOFA, sequential organ failure assessment scoring; APACHE, acute physiology and chronic health evaluation scoring.

**Figure 5:**
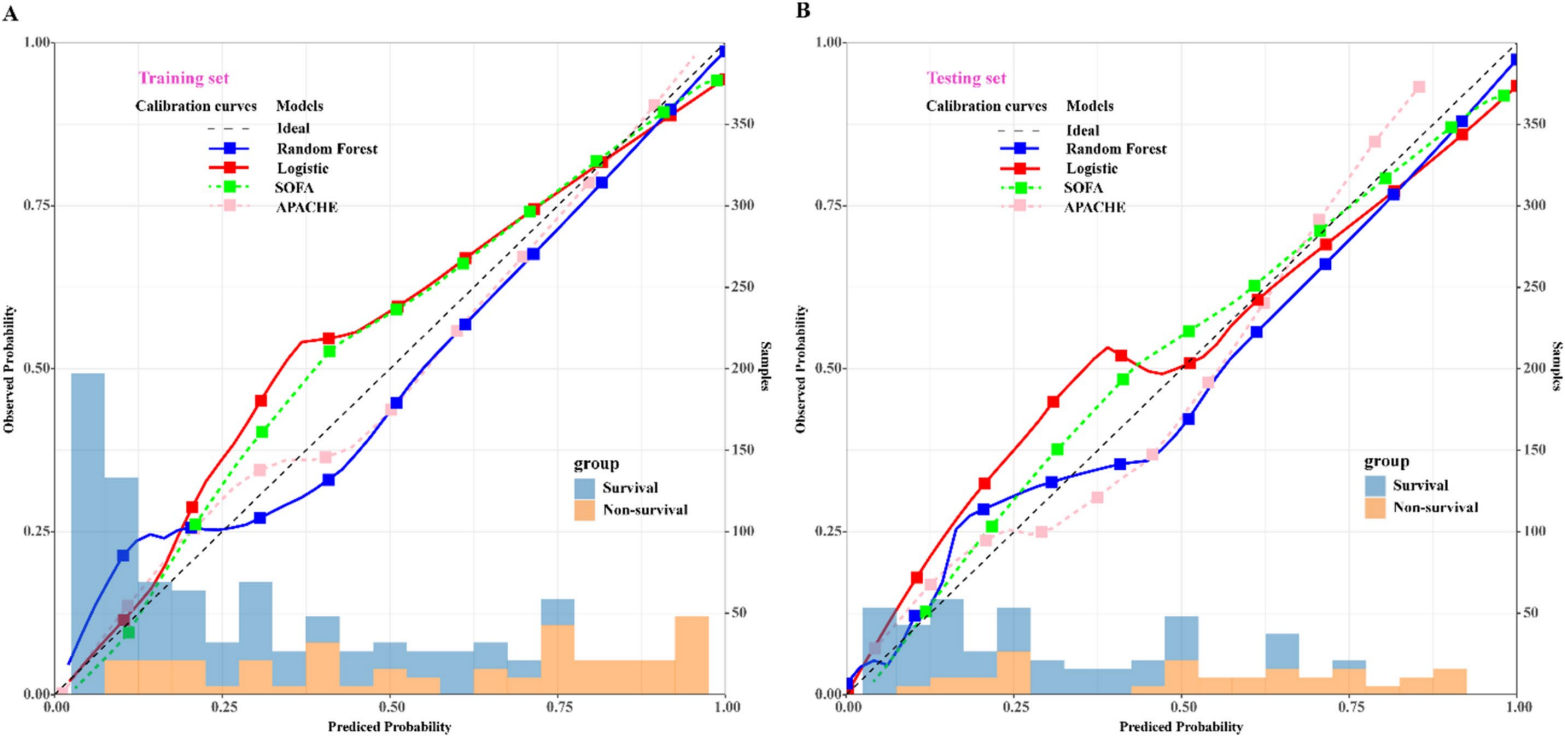
Comparison of calibration curves among RF, logistic regression, SOFA, and APACHE scoring system. **(A)** Training set; **(B)** validation set. The blue solid calibration curves which were notably closer to the ideal reference line both in training set and validation set represented that RF associated with the best goodness-of-fit and accuracy of prediction among the four models. SOFA, sequential organ failure assessment scoring; APACHE, acute physiology and chronic health evaluation scoring. The left *x-axis* represents the observed probability; the right *x*-axis represents the sample size, *y*-axis represents the predicted probability.

**Figure 6:**
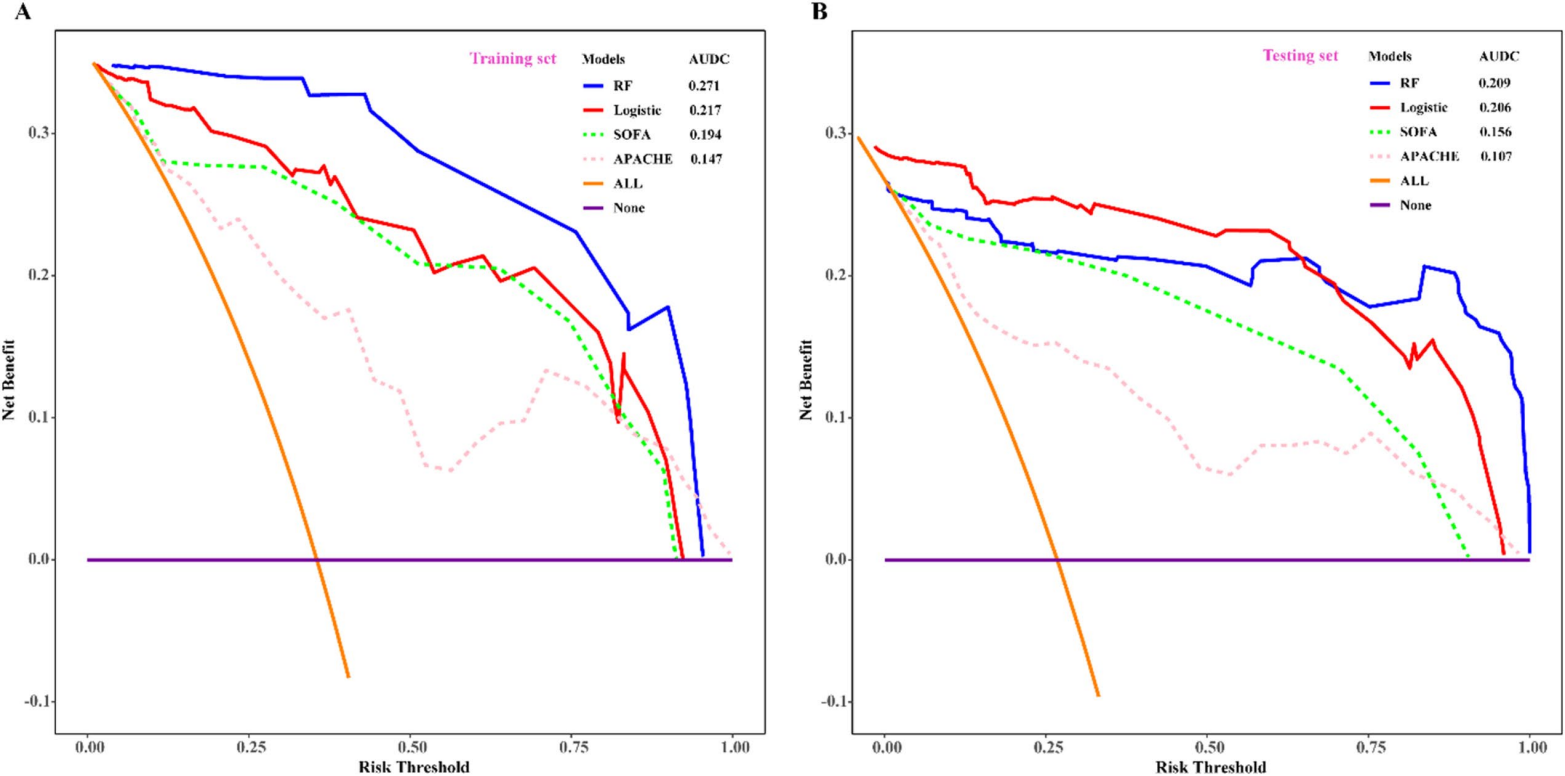
Comparison of decision curve analysis among RF, logistic regression, SOFA, and APACHE scoring system. **(A)** Training set; **(B)** validation set. With the highest value of AUDC and net benefit both in training set and validation set, RF was considered as the optimum model which associated with the best clinical practicality. SOFA, sequential organ failure assessment scoring; APACHE, acute physiology and chronic evaluation scoring. AUDC, area under DCA curve.

To assess the predictive efficacy of traditional logistic and RF models, we conducted assessments of discrimination, calibration, and clinical net benefits. Additionally, we compared the performance of logistic regression and RF models with SOFA (4) and APACHE (6) to explore clinical practicality. Discrimination results indicated that the among the predictive models of RF, logistic, SOFA, and APACHE, the AUCs and their corresponding 95% confidence intervals (CIs) were significantly larger (*P*_Delong’s test_ < 0.05) in both training and validation sets compared to other three models (Figures 4A,B). For model calibration, we observed that calibration curves of RF were notably closer to the ideal reference line compared to other models in both training and validation sets, which indicated that comparing to other models, the RF model associated with better fitting goodness and predictive ability (Figures 5A,B). Results of clinical practicality, as indicated by the Area Under Decision Curve (AUDC), showed that in the training set (Figure 6A) and validation set (Figure 6B), comparing with other three models, the AUDCs of RF model were with the highest values. These findings illustrated that the RF model yielded optimal clinical net benefit for predicting mortality in adult sepsis patients.

### Variables importance of logistic and RF models

The variable importance calculations from both the logistic regression and RF models are presented in Supplementary Figure S2. In predicting mortality in the adult sepsis cohort, the logistic regression model identified systolic pressure, lactic acid, IL6, and NLR as the most important variables, followed by Tbil, PT, and RDW. Consistently, the RF model also highlighted systolic pressure, lactic acid, IL6, and NLR as the most crucial variables for predicting mortality. However, the variables with relatively less importance in the RF model were RDW, NEUT, and Tbil, in contrast to the logistic regression model.

## Discussion

In this study, we investigated the risk factors predicting the mortality of adult patients with sepsis, employing both the traditional logistic regression approach and the RF approach. Overall, both models yielded similar results, with only slight differences in the included variables, with the inclusion of PT as a risk factor in the logistic regression model, while NEUT was included in the RF model. To assess the predictive capabilities of these models for adult sepsis prognosis, we conducted comprehensive validations, considering discrimination, calibration, and clinical benefits. Among the three above criterion of model assessment, calibration is a critical aspect of evaluating the performance of clinical prediction models. It refers to the degree to which the predicted probabilities of an event match the actual observed outcomes. A well-calibrated model is one where the predicted probabilities are reliable indicators of the likelihood of the event occurring in practice. This is particularly important in clinical settings, where accurate predictions can guide treatment decisions and patient management. Additionally, we compared the models with the widely used SOFA and APACHE scoring systems based on these criteria. The results of model validation and comparison demonstrated that the RF model exhibited significant superiority over the logistic regression model, as well as over the SOFA and APACHE scoring systems, in predicting mortality in adult sepsis patients.

### Application of biomarkers in adult sepsis prediction

Sepsis represents an aberrant inflammatory response triggered by pathogenic microorganism infection. There is an increasing consensus suggesting that the immune system’s activation in the early stages and its subsequent inhibition in the later stages can both contribute to alterations in circulating levels of inflammatory mediators (24–26). While the exact mechanisms of sepsis remain incompletely understood, studies have highlighted the crucial role of biomarkers in sepsis diagnosis and prognosis prediction, significantly impacting the risk of mortality (27–29). Our study exhibited that besides systolic pressure, biomarkers such as lactic acid, RDW, NLR, IL6, NEUT, and Tbil were incorporated into the traditional logistic and RF models we constructed. A closer examination through variable importance analysis revealed that lactic acid, NLR, and IL6 played pivotal roles in determining the significance of variables in both models.

Lactic acid, a metabolic byproduct of anaerobic glucose fermentation, poses a threat to the human body when present at elevated levels. High concentrations of lactic acid not only inhibit the activity of various essential enzymes but also mitigate the sensitivity of endothelial cells to vasoactive drugs (30). Furthermore, in the context of microbial infection or sepsis, lactic acid assumes a critical role in suppressing immune cells, potentially leading to immune suppression and severe consequences for the individual (31). Elevated levels of lactic acid in patients with sepsis are associated with poor outcomes, as they reflect inadequate perfusion and oxygen delivery to tissues. Studies have shown that high lactate levels correlate with increased mortality rates in septic patients, making it a valuable prognostic marker. Over the years, numerous studies have underscored the association between elevated lactic acid levels and increased mortality rates in sepsis (4, 32, 33).

The NLR serves as a biomarker calculated by the ratio of neutrophil to lymphocyte counts, encompassing both the innate immune response, primarily mediated by neutrophils, and adaptive immunity, supported by lymphocytes (34). Neutrophils act as the frontline defenders against pathogen invasion through processes like chemotaxis and phagocytosis. Upon activation by pathogens, various cytokines, granular proteins, and reactive oxygen species (ROS) are produced and released by neutrophils (35). While this activation is crucial for pathogen resistance, excessive activation leading to increased production of ROS and cytokines may damage vascular endothelial cells through different mechanisms, resulting in tissue hypoperfusion and life-threatening organ failure (36). Consequently, an elevated neutrophil count, or a decreased lymphocyte count, contributes to an increased NLR, serving as a predictor of disease severity and poor prognosis in various conditions such as severe trauma (37), stroke (38), malignant tumor (39, 40) and sepsis (41, 42). Previous studies on NLR in predicting sepsis prognosis have demonstrated its independent association with high in-hospital mortality rates, showcasing significant advantages over conventional scores like SOFA or APACHE (43, 44). In summary, NLR stands out as a valuable biomarker for predicting mortality in sepsis patients.

Pro-inflammatory cytokines play a critical role in sepsis pathogenesis. IL-6, a member of the 4-helical cytokine family, activates signaling pathways by binding to an 80-kDa cytokine receptor (IL-6R). IL-6 plays a pivotal role in the immune response to infection, and it is released by various cells, including macrophages and T cells, in response to inflammatory stimuli. During sepsis, IL-6 is produced in response to pathogenic stimuli, and IL-6R is generated by neutrophils. Consequently, the IL-6/IL-6R complex triggers the phosphorylation and redistribution of VE-cadherin, leading to vascular endothelial damage and leakage (45). Excessive vascular endothelial damage and leakage in sepsis patients can result in blood pressure decline, hemodynamic collapse, irreversible septic shock, and even death. Clinical predictive models have consistently shown that IL-6 holds favorable predictive value for sepsis severity and prognosis. Elevated levels of IL-6 suggest severe illness and poor prognosis (46, 47). Moreover, studies have indicated that immunotherapeutic blockade of IL6 could reduce the mortality rate in sepsis (48).

### Application of advanced statistical methods to complement common approaches

The RF algorithm possesses numerous statistical and computational advantages. This algorithm employs integrated learning, wherein its fundamental component is typically a decision tree, placing it within the broader category of integrated learning methods (49, 50). The terminology “random” and “forest” in RF signifies the amalgamation of classifiers, where each tree functions as an individual classifier. Notably, RF operates with hundreds of trees in parallel, collectively forming a forest. RF consolidates the results of all classification votes, designating the category with the highest votes as the final output, aligning with the Bagging concept and reflecting the core idea of RF (51). In contrast to the traditional logistic regression algorithm, RF demonstrates several distinct advantages: (1) RF employs an integrated algorithm with exceptionally high accuracy; (2) The randomness in model construction reduces susceptibility to overfitting; (3) It can handle discrete, continuous, or high-dimensional data without requiring data normalization; (4) The OOB feature allows obtaining unbiased estimates of true errors during model generation without losing training data. In the present study, the RF model demonstrated its superiority in predicting the prognosis of adult sepsis, exhibiting better discrimination, calibration, and clinical decision-making compared to traditional statistical methods (52, 53). Although RF model improves prediction accuracy by integrating multiple decision trees, but this also makes their decision-making process relatively complex and difficult to explain. Each decision tree is trained based on a randomly selected subset of features, which increases the model’s diversity but also makes it challenging to interpret. So as to address these limitations, we can solve these problems by conducting feature importance analysis, visualizing individual decision trees, employing local explainability methods, and integrating doctors’ experiences and expertise, it is possible to address the limitations of interpretability to a certain extent.

However, the present study has limitations that should be acknowledged. Firstly, being retrospective and cross-sectional, it relies on some laboratory results reflecting the patient’s condition at specific time points, which may not be generalizable to the entire population. We expect to validate the current research and strengthen the impact of this study through prospective research. Secondly, we explicitly state that our initial predictor selection was based on univariate analysis, which may not capture the full complexity of the relationships between the predictors and the outcome variable. Thirdly, despite RF’s significant advantages in predicting sepsis mortality compared to traditional regression, its interpretability limitation remains noteworthy. Finally, due to this is a single center study and without testing on an independent dataset, the model’s accuracy could be artificially inflated, reducing its generalizability. Therefore, integrating RF with traditional regression approaches could enhance the predictive capabilities of healthcare research in the future.

## Conclusion

In conclusion, logistic regression and RF models were developed to predict mortality in adult sepsis patients, with both models identifying consistent risk factors. The RF model outperformed traditional regression and the SOFA and APACHE scoring systems, highlighting its superiority in mortality prediction.

## Data Availability

The original contributions presented in the study are included in the article/Supplementary material, further inquiries can be directed to the corresponding authors.
