# Supplementary material for: Comparison between traditional logistic regression and machine learning for predicting mortality in adult sepsis patients
Source: Front Med (Lausanne). 2025 Jan 6;11:1496869. doi: 10.3389/fmed.2024.1496869 (PMC11743956; doi:10.3389/fmed.2024.1496869)
Supplement: Supplementary file 1 [file Data_Sheet_1.docx]

**Supplementary materials of “Comparison between Traditional Logistic Regression and Random Forest via Machine Learning for Predicting Mortality in Adult Sepsis Patients”**


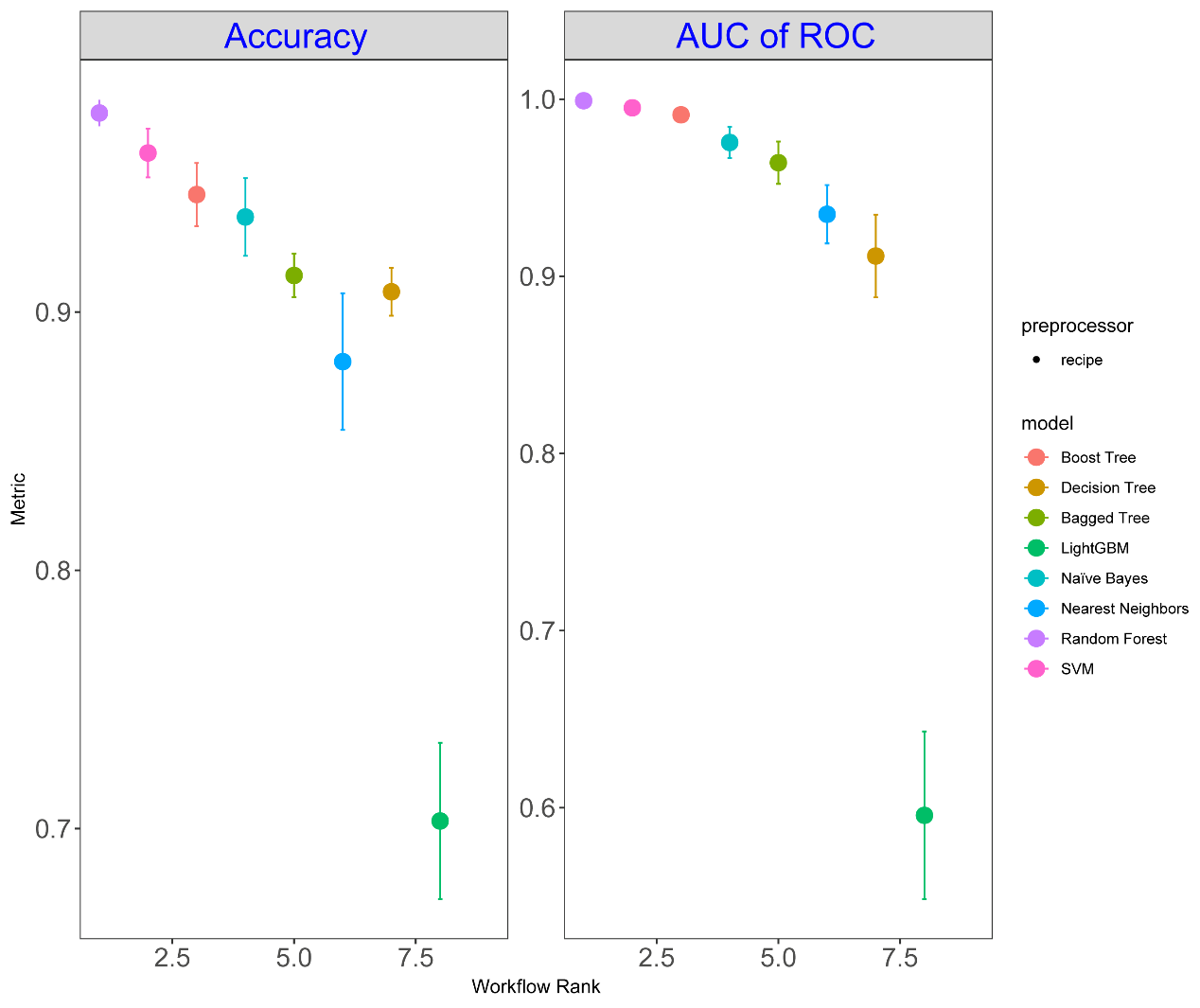


**Supplementary Fig1:** Machine learning models selection. With the largest AUC of ROC and accuracy value, random forest was selected as machine learning model for this study.


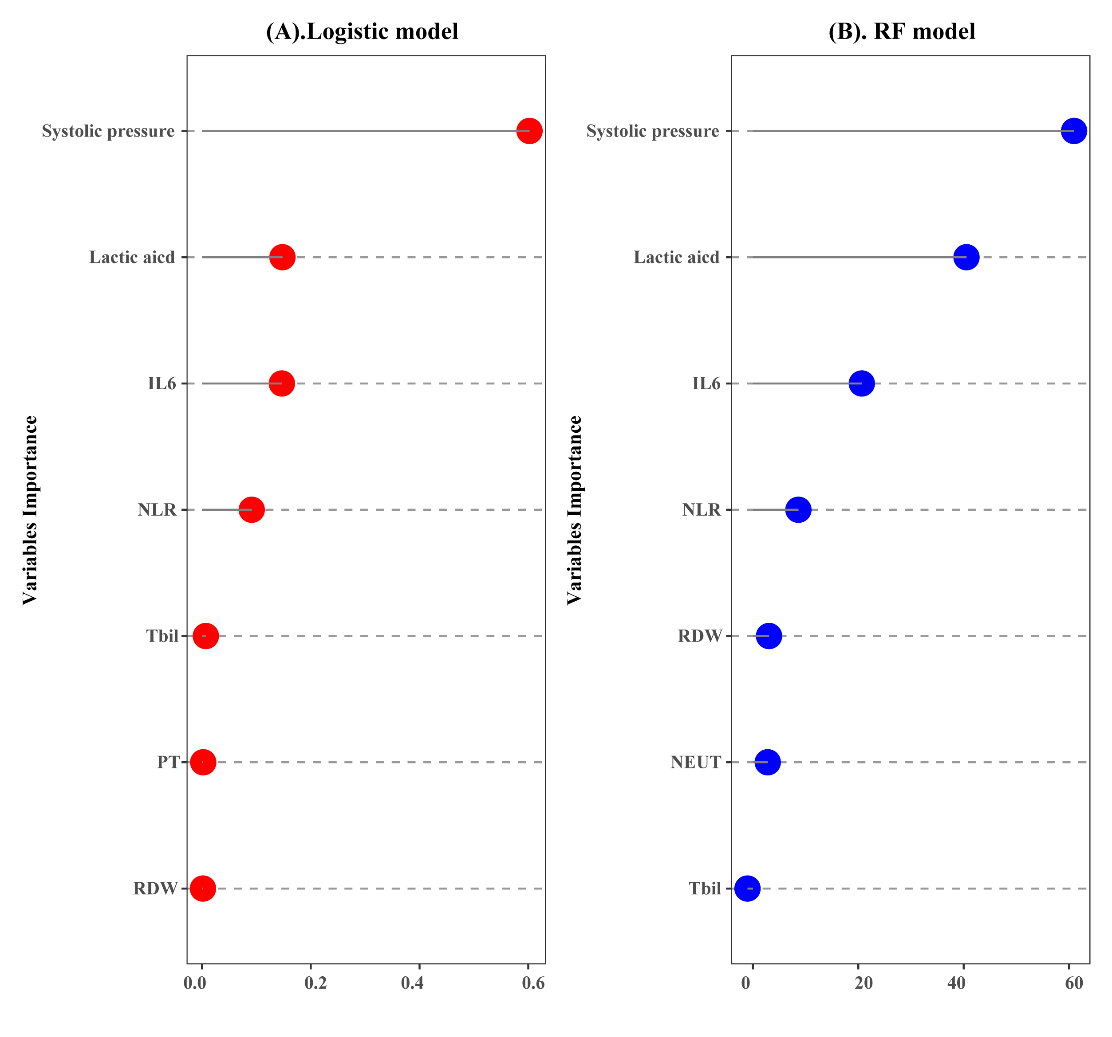


**Supplementary Fig2:** Variables importance of Logistic and random forest models.

**Supplementary Tbale1: Logistic model selection by step regression via the least AIC**

| **Model** | **OR** | **95%CI** | **p value** | **AIC** |
| --- | --- | --- | --- | --- |
| Model1 | 0.009 | 0.001-6.096 | 1.194 | 115.22 |
| Model2 | 0.008 | 0.001-3.882 | 1.152 | 113.24 |
| Model3 | 0.008 | 0.001-3.826 | 1.150 | 111.30 |
| Model4 | 0.005 | 0.001-1.953 | 1.100 | 108.21 |
| Model5 | 0.009 | 0.001-2.009 | 1.115 | 108.20 |
| Model6 | 0.015 | 0.001-2.791 | 1.147 | 106.90 |
| Model7 | 0.012 | 0.001-2.073 | 1.119 | 105.90 |
| Model8 | 0.015 | 0.002-2.479 | 1.017 | 103.20 |
| Model9 | 0.013 | 0.003-2.312 | 1.003 | 102.33 |
| Model10 | 0.561 | 0.021-1.118 | 0.982 | 100.12 |
| Model_Logistic_ | 1.012 | 2.218-3.216 | 0.014 | 98.65 |

Model1=Gender+CHD+Systolic pressure+WBC+NEUT+LYMP+

Lactic acid+NLR+RDW+IL6+ PT+INR+FBI+D-dimer+AST+

Tbil+ Lung infection

Model2=Model1-Gender

Model3=Model2-CHD

Model4=Model3-WBC

Model5=Model4-NEUT

Model6=Model5-LYMP

Model7=Model6-INR

Model8=Model7-FBI

Model9=Model8-D-dimer

Model10=Model9-AST

Model_Logistic_=Model10-Lung infection
